# Genome-Wide Identification of Long Non-coding RNAs in the Gravid Ectoparasite *Varroa destructor*

**DOI:** 10.3389/fgene.2020.575680

**Published:** 2020-10-16

**Authors:** Zheguang Lin, Yibing Liu, Xiaomei Chen, Cong Han, Wei Wang, Yalu Ke, Xiaoling Su, Yujiao Li, Heng Chen, Hao Xu, Guohong Chen, Ting Ji

**Affiliations:** ^1^Apicultural Research Institute, College of Animal Science and Technology, Yangzhou University, Yangzhou, China; ^2^Wuzhong Animal Health Supervision Institute, Suzhou, China; ^3^Jinhua Academy of Agricultural Sciences, Jinhua, China; ^4^Shandong Apiculture Breeding of Improved Varieties and Extension Center, Tai’an, China

**Keywords:** *Varroa destructor*, long non-coding RNA, honey bee, ectoparasitic mite, oogenesis

## Abstract

Long non-coding RNAs (lncRNAs) emerge as critical regulators across a wide variety of biological functions in living organisms. However, to date, no systematic characterization of lncRNAs has been investigated in the ectoparasitic mite *Varroa destructor*, the most severe biotic threat to honey bees worldwide. Here, we performed an initial genome-wide identification of lncRNAs in *V. destructor* via high-throughput sequencing technology and reported, for the first time, the transcriptomic landscape of lncRNAs in the devastating parasite. By means of a lncRNA identification pipeline, 6,645 novel lncRNA transcripts, encoded by 3,897 gene loci, were identified, including 2,066 sense lncRNAs, 2,772 lincRNAs, and 1,807 lncNATs. Compared with protein-coding mRNAs, *V. destructor* lncRNAs are shorter in terms of full length, as well as of the ORF length, contain less exons, and express at lower level. GO term and KEGG pathway enrichment analyses of the lncRNA target genes demonstrated that these predicted lncRNAs may be potentially responsible for the regulatory functions of cellular and biological progresses in the reproductive phase of *V. destructor*. To our knowledge, this is the first catalog of lncRNA profile in the parasitiformes species, providing a valuable resource for genetic and genomic studies. Understanding the characteristics and features of lncRNAs in *V. destructor* would promote sustainable parasite control.

## Introduction

Colony losses of the western honey bee *Apis mellifera* in the Western world are a serious issue due to the critical role of honey bees in the balance of the ecosystem, sustainable agriculture, and food security (Neumann and Carreck, 2010; Potts et al., 2016; Steinhauer et al., 2018). There has been a consensus that an emerging ectoparasitic mite, *Varroa destructor*, is the principal threatening factor (Neumann and Carreck, 2010; Nazzi and Conte, 2016; Evans, 2019). *V. destructor* feasts on the fat body (Ramsey et al., 2018, 2019) of honey bees, transmits viruses, and affects host immunity (Yang and Cox-Foster, 2005; Rosenkranz et al., 2010; Di Prisco et al., 2016), severely interrupting the social organization and demographic continuity in *A. mellifera* colonies. Without treatment against this mite, infested *A. mellifera* colonies usually die within 6 months to 2 years (Kraus and Page, 1995; Le Conte et al., 2010). In the wake of the occurrence of *V. destructor* in New Zealand in 2000 (Todd et al., 2007; Mondet et al., 2014) and in Hawaii in 2007 (Martin et al., 2012), this mite has been distributed globally except for Australia and a few remote islands (Muli et al., 2018; Noël et al., 2020; Traynor et al., 2020).

*V. destructor* lives entirely on its host and cannot survive independently (Traynor et al., 2020) with two life cycles: the phoretic (non-reproductive) phase on the body surface of adult bees and the reproductive phase in the sealed brood cells with immature bees (Martin, 1994). Reproduction of the parasite starts from the oogenesis process, which occurs since approximately 6 h later after the invaded cell is capped and is crucial for understanding the reproductive biology of the parasite (Garrido et al., 2000; Häußermann et al., 2019). A great number of studies have been performed on different life phases of this obligate bee parasite, however, molecular studies are very limited as a result of the lack of genomic information. Cornman et al. (2010) analyzed *V. destructor* genome sequence for the first time, which largely facilitated Mondet et al. (2018) to investigate a full life cycle transcriptomic profiling in adult *V. destructor*. Nevertheless, the emerging functional elements of non-coding RNAs (ncRNAs) have yet to report with this parasitic mite.

Numerous genome-wide transcriptome has observed that the majority of transcripts do not code for proteins, and these transcripts are referred to as ncRNAs (Lee et al., 2012). NcRNA is a generic term for all functional RNAs that are transcribed from DNA but not translated into proteins, and the widespread expression of ncRNAs in eukaryotic cells has been demonstrated to be of importance in the processes of development, disease resistance, etc. (Esteller, 2011). Long non-coding RNAs (lncRNAs), most of which are located in the nucleus of eukaryotes, are a cluster of ncRNAs with a length of more than 200 nt, with cap-structure and ploy (A)-tail but usually without a long reading frame (Guttman et al., 2009). LncRNAs can be classified into four groups based on their positional information on genomes, i.e., sense lncRNAs, intergenic lncRNAs (lincRNAs), intronic lncRNAs (ilncRNAs), and antisense lncRNAs (lncNATs) (Harrow et al., 2012; Ma et al., 2013). As functional elements, lncRNAs have been proved to exert their bioactivities by regulating gene expression at the level of epigenetic inheritance, transcription, and post transcription, as well as by affecting protein localization and telomere replication (Furuno et al., 2006; Mercer et al., 2009). Currently, however, studies of lncRNAs in the field of honey bee science are still in its infancy.

The present a few studies have demonstrated that these functional elements of lncRNAs participate in the regulation of the physiological processes of honey bees, such as labor division, ovary development, neural networks, pesticide metabolism, and pathogen resistance (Kiya et al., 2008; Humann et al., 2013; Jayakodi et al., 2015; Chen et al., 2017, 2019; Liu et al., 2019; Fent et al., 2020). Guo et al. (2018a, b) screened the lncRNAs in two honey bee fungi, *Ascospheara apis* and *Nosema ceranae*, providing the first two lncRNA profiles in honey bee pathogenic agents. In the present study, we deeply sequenced the ubiquitous ectoparasite *V. destructor* on the Illumina platform during the oogenesis stage. We identified 6,645 novel lncRNA transcripts corresponding to 3,897 lncRNA genes in the detrimental mite. The subcellular localization patterns of the lncRNAs were predicted, most of which were located in nucleus. The structural features and the subcellular localization of the lncRNAs identified in this study showed consistent with their counterparts in the mammals. In order to comprehensively investigate the main biological properties and functions of the target genes of the putative novel lncRNAs, we performed Gene Ontology (GO) and pathway enrichment analyses. By comprehensively identifying lncRNAs in the gravid *V. destructor*, we aimed to offer novel insights into understanding the basic molecular biology of this ubiquitous ectoparasitic mite of honey bees.

## Materials and Methods

### Gravid Adult Female *Varroa destructor* Mite Collection

*V. destructor* mites were collected from experimental *A. mellifera* colonies untreated for half a year, which were located in Yangzhou University of China. We employed the mites to mimic phoretic phase before experimental infestation to obtain similar physiological status (see details in Lin et al., 2018). Each freshly capped worker cell was introduced with a mite, and gravid adult mites were collected from sealed worker brood with the tweezers and the paint brushes 2 days later after infestation (Supplementary Figure 1). Three samples, each of which contained 15 randomly gathered mites, were prepared for RNA-seq (i.e., Vd-1, Vd-2, and Vd-3; Table 1). All the mites were frozen in liquid nitrogen after bathing in the phosphate buffered saline, pH 7.4 (Sigma, MO, United States), twice. The samples were stored in −80°C until RNA extraction.

**TABLE 1:** Throughput and quality of RNA-seq of the three libraries.

Sample name	Total nucleotides (G)	Raw reads	Clean reads	Q20 (%)	Q30 (%)	GC content (%)
Vd-1	13.3	88,932,536	88,072,510	97.3	92.2	43.6
Vd-2	12.6	84,224,988	83,475,384	97.3	92.3	43.7
Vd-3	16.8	111,930,228	108,997,328	97.7	93.5	43.0

### Library Preparation for lncRNA Sequencing

Total RNA was extracted using TRIzol reagent kit (Invitrogen, CA, United States) according to the manufacturer’s protocol. Purity and quantity of total RNA were measured using NanoDrop^TM^ 2000 (Thermo Fisher Scientific, DE, United States). RNA quality was assessed using the RNA Nano 6000 Assay Kit of the Bioanalyzer 2100 system (Agilent Technologies, CA, United States) and was checked on RNase free agarose gel electrophoresis. For the sample preparation, 3 μg RNA was used for each of the three *V. destructor* samples, and NEBNext^®^ UltraTM RNA Library Prep Kit for Illumina^®^ (NEB, MA, United States) was used to generate sequencing libraries. After removing rRNAs, the enriched mRNAs and ncRNAs were fragmented into short fragments by using fragmentation buffer and reverse transcribed into cDNA with random primers. Second-strand cDNA were subsequently synthesized by using DNA polymerase I, RNase H, dNTP, and buffer. The cDNA fragments were then purified with QiaQuick PCR extraction kit (Qiagen, Venlo, The Netherlands), end repaired, poly(A) added, and ligated to Illumina sequencing adapters. Uracil-N-Glycosylase was used to digest the second-strand cDNA and the digested products were size selected by agarose gel electrophoresis, PCR amplified, and sequenced on an Illumina HiSeq^TM^ 2500 platform at the Novogene Bioinformatics Institute (Beijing, China). The raw sequencing data were uploaded to the National Centre for Biotechnology Information (SRA accession: SRP258850).

### LncRNA Identification and Analyses

Clean reads were obtained by removing reads containing adapter, or ploy-N and low-quality reads from the raw data. Meanwhile, we calculated the Q20, Q30, and GC content. The paired-end clean reads were mapped to the reference genome of *V. destructor*^1^ (Techer et al., 2019) using HISAT2 (Kim et al., 2015). We then reconstruct the transcripts with StringTie and HISAT2. To identify new transcripts, all the reconstructed transcripts were aligned to the reference genome and were divided into 12 categories by employing Cuffcompare, and transcripts with one of the five class codes “I,” “j,” “o,” “u,” and “x” were potentially recognized as novel ones. The putative novel transcripts were further eliminated by removing the ones with length ≤ 200 nt or with exon number <2. Coding Potential Calculator (CPC), Coding-Non-Coding Index (CNCI), and Pfam-scan (PFAM) were jointly used to assess the protein-coding potential of the selected novel transcript candidates and the intersection was considered as the candidate set of lncRNAs. We used StringTie again to quantify transcripts abundances by calculating the FPKM (expected fragments per kilobase of transcript per million fragments mapped) values. All the putative novel lncRNAs were computed their subcellular localization by means of lncLocator, an online software for lncRNA location prediction based on a stacked ensemble classifier (Cao et al., 2018). LncLocator can predict five subcellular localizations of lncRNAs, including nucleus, cytoplasm (part of cytoplasm except for cytosol, ribosome, and exosome), ribosome, cytosol, and exosome. Then, we searched coding genes 100 kb upstream and downstream of the predicted novel lncRNAs as *cis* target genes (Guil and Esteller, 2012; Feng et al., 2019), which were subjected to enrichment analysis of GO functions and kyoto encyclopedia of genes and genomes (KEGG) pathways.

The statistical analyses (Student’s *t*-test) of the characteristic differences between lncRNAs and mRNAs were performed with SPSS Statistics 25.

### RT-PCR Validation

Removal of gDNA and synthesis of cDNA was performed with RNA products following the manufacturer’s instructions of ReverTra Ace qPCR RT Master Mix (Tiangen, Beijing, China). To validate the putative lncRNAs in *V. destructor* mites, 16 lncRNAs were randomly selected to determine with PCR amplification, which was carried out with the obtained cDNA in a 20 μL reaction volume mixture (2 × Taq PCR StarMix; GenStar, Beijing, China) on an Eppendorf cycler. PCR profile consists of a pre-denaturation at 94°C for 5 min; followed by 30 cycles including 94°C for 50 s, 55°C for 30 s, and 72°C for 1 min; and a final elongation step at 72°C for 10 min (Guo et al., 2018a). The tested lncRNAs with their forward and reverse primers were presented in Supplementary Table 1. PCR products were electrophoresed in 2.5% Tris acetate-EDTA-agarose gel containing 0.01% Gelview (BioTeke, Beijing, China) and visualized under ultraviolet light (Peiqing, Shanghai, China).

## Results

**FIGURE 1:**
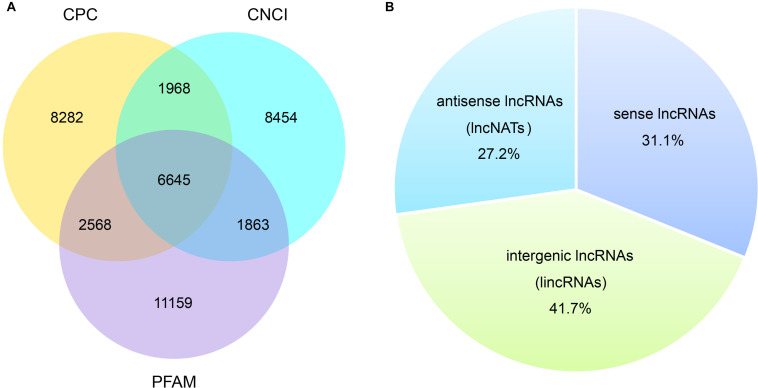
Prediction of novel lncRNA of *V. destructor*. **(A)** Venn analysis of the putative novel lncRNAs by using CPC, CNCI, and PFAM. **(B)** The source and distribution percentage of the novel lncRNAs based on the intersection of Venn diagram.

Total RNA of the three *V. destructor* samples (Vd-1, Vd-2, and Vd-3) were isolated and sequenced. Overall, 42.8 G sequencing data were generated, corresponding to 285.1 million raw reads and 280.5 million quality filtered (clean) reads were generated from the three cDNA libraries (Table 1). G20, G30, and GC content were also shown in Table 1 with the mean values of 97.4, 92.7, and 43.5%. For the three *V. destructor* samples, 93.2, 93.6, and 90.0% obtained reads were mapped to the reference genome sequence, and the mapped regions of each sample on the genome were shown in Supplementary Figure 2. Then 6,645 putative novel non-coding transcripts were predicted using CPC, CNCI, and PFAM (Figure 1A and Supplementary Table 2), of which 2,066 (31.1%) were sense lncRNAs, 2,772 (41.7%) were lincRNAs, and 1,807 (27.2%) were lncNATs (Figure 1B). IlncRNAs were not observed in this mite. In addition, 32,415 protein coding transcripts were obtained, with 32,331 mapped to the reference genome, and the remaining 84 ones were not annotated (Supplementary Table 2).

**FIGURE 2:**
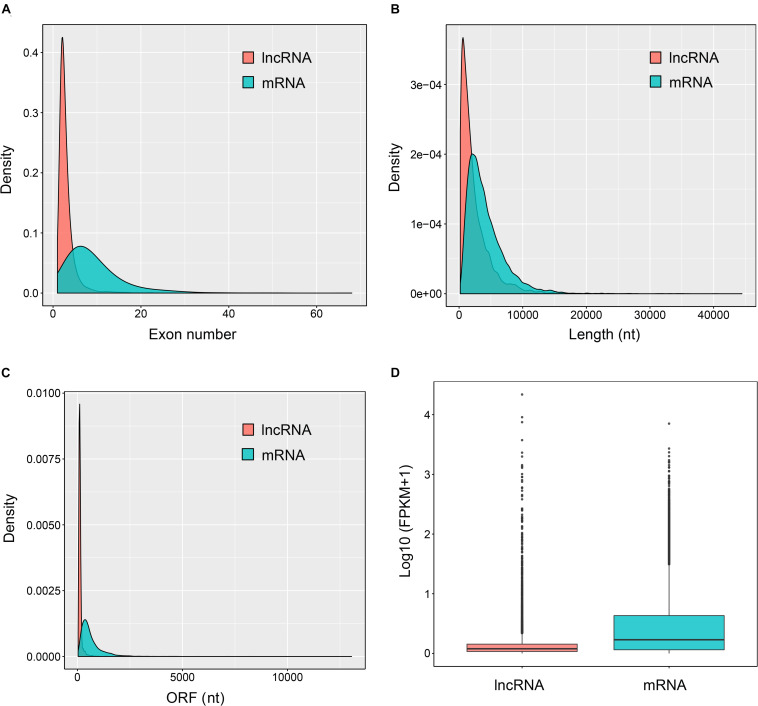
Genomic features of the predicted lncRNAs. Exon number distribution **(A)**, length distribution **(B)**, ORF length distribution **(C)**, and expression level indicated by log10 (FPKM + 1) **(D)** of 32,415 coding transcripts (mRNAs) and 6,645 novel lncRNAs were plotted.

Most lncRNAs contained two exons (55.8%), followed by three (23.8%), four (9.6%), five (4.2%), six (2.2%), and seven (1.1%) (Figure 2A and Supplementary Table 2). The ratio of lncRNAs is less than one when the number of exons is greater than seven, and they contained at most 27 exons (Figure 2A and Supplementary Table 2). This was significantly different from the coding transcripts (Student’s *t*-test, *p* < 0.001), which peaked at six exons (9.5%) and were up to 68 exons. Meanwhile, the ratio of mRNAs with three to eight exons were respectively more than seven (Figure 2A and Supplementary Table 2). Most of both lncRNAs (55.4%) and mRNAs (65.5%) ranged from 1,000 to 5,000 nt in length. But then, 33.2% lncRNAs were less than 1,000 nt and 24.7% mRNAs were between 5,001 nt and 10,000 nt. For the long sequence (>10,000 nt), 2.1% lncRNAs and 4.7% mRNAs were occupied. As a result, lncRNAs averaged 2,435 nt in length, which was significantly shorter than protein-coding genes (4,187 nt; Student’s *t*-test, *p* < 0.001; Figure 2B and Supplementary Table 2). Regarding the length of open reading frames (ORFs) in lncRNAs and mRNAs, we got a similar trend with above. Most of the ORFs of both lncRNAs (58.5%) and mRNAs (51.4%) were in the middle range 100–500 nt in length, followed by 39.9% lncRNAs ≤100 nt and 31.9% mRNAs ranging from 501 to 1,000 nt. Consistently, 0.2% lncRNAs and 16.3% mRNAs were respectively greater than 1,000 nt, and the mean length of ORFs in lncRNAs was significantly shorter than that of mRNAs (132 vs. 649 nt; Student’s *t*-test, *p* < 0.001; Figure 2C and Supplementary Table 2). Further, the expression level of lncRNAs showed significantly lower compared to mRNAs (Figure 2D). Additionally, 16 lncRNAs were randomly chosen to be validated with RT-PCR with 15 (93.8%) successful amplification (Supplementary Figure 3). Sanger sequencing confirmed most of the validated lncRNAs (Supplementary Table 5) although two of the 15 failed due to the low expression, which were reflected by the dimmed electrophoretic band in Supplementary Figure 3 (band 2 and 4).

**FIGURE 3:**
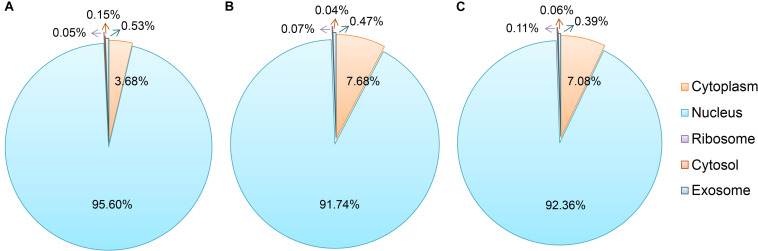
Subcellular localization prediction of the sense lncRNAs **(A)**, the intergenic lncRNAs (lincRNAs, **B**), and the antisense lncRNAs (lncNATs, **C**). An online prediction program, lncLocator, which can predict five subcellular localizations of lncRNAs, was used for this analysis. The term “cytoplasm” here includes the part of cytoplasm except for cytosol, ribosome, and exosome.

**FIGURE 4:**
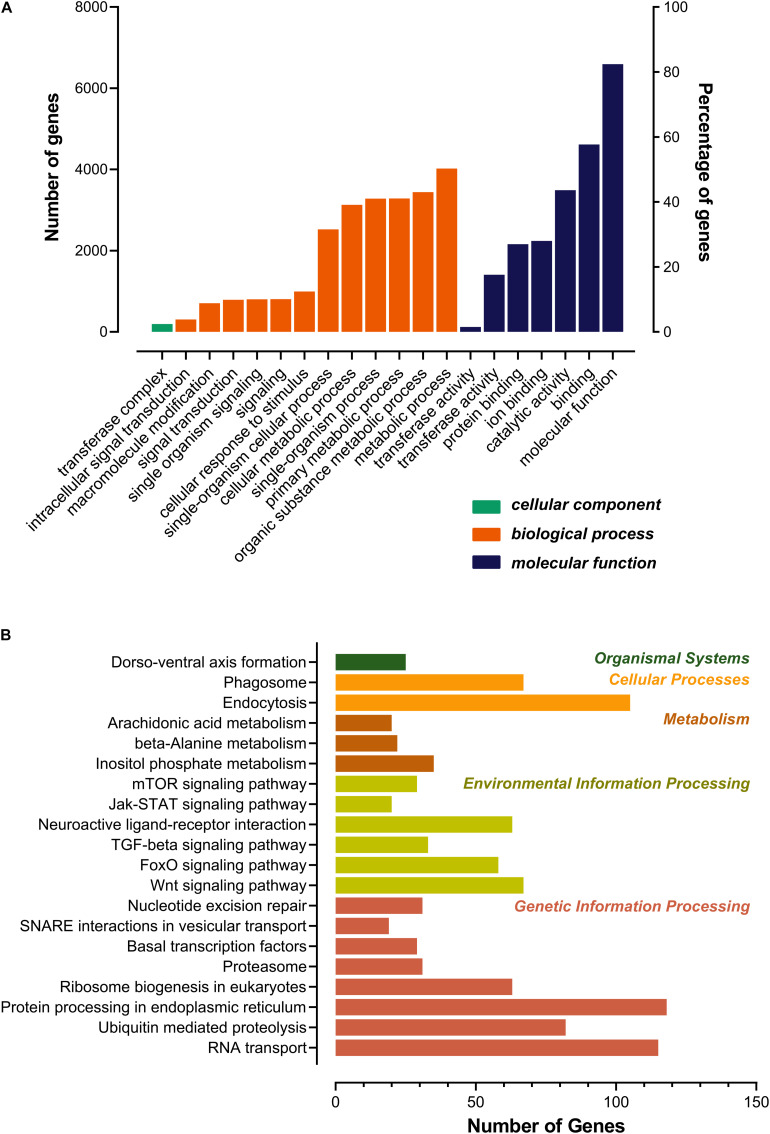
Gene ontology (GO) categorization **(A)** and kyoto encyclopedia of genes and genomes (KEGG) pathway **(B)** analyses for the target genes of the predicted lncRNAs. Top 20 enriched terms were respectively shown. **(A)** Genes were assigned to three GO categories: cellular component, biological process, and molecular function. **(B)** The pathway terms were classified as the main functions. The color of each bar corresponded to the right specific color of the function form, which referred to the KEGG pathway map (https://www.genome.jp/kegg/kegg2.html).

As shown in Figure 3, most of the novel lncRNAs were predicted to be localized in the nucleus, followed by in cytoplasm, irrespective of their different sources. In total, only 31, 5, and 5 lncRNAs were predicted in exosome, cytosol, and ribosome, respectively. We then obtained 9,500 target genes of novel lncRNAs in *cis* regulation. GO term analysis indicated the target genes were in the ontology class of cellular component, molecular function, and biological process (Figure 4A and Supplementary Table 3). The top five enriched GO terms with extremely small padj values participated in protein binding, catalytic activity, and transferase activity, indicating that lncRNAs may play roles in the important process of enzyme-related catalytic activities in *V. destructor* (Figure 4A and Supplementary Table 3). The KEGG pathway enrichment denoted that the target genes mostly participated in 104 pathways (Supplementary Table 4). The top 20 enriched pathways, which was divided into five classes based on their major functions, i.e., organismal systems, cellular processes, metabolism, environmental information processing and genetic information processing, were displayed in Figure 4B. The top five enriched pathways were endocytosis, RNA transport, phagosome, ubiquitin mediated proteolysis, and protein processing in endoplasmic reticulum (Supplementary Table 4), involving the critical processes of endocytosis, and protein processing and degradation, which belong to the cellular processes and the genetic information processing (Figure 4B). Notably, 115 genes are enriched in the RNA transport, which is a fundamental process for gene expression, signifying lncRNAs may play key parts in the transcriptional regulation during the oogenesis of *V. destructor*.

## Discussion

LncRNAs have emerged as critical participators in a variety of cellular activities, ranging from simple housekeeping to complex regulatory functions. However, till now, the studies of lncRNAs are mainly conducted in the field of humans, mammal, and crops. In contrast, the research on invertebrates is still at the early stage. Here, we identified the sequences and expression features of the lncRNAs via high-throughput sequencing technology, for the first time, in a devastating ectoparasite, *V. destructor*, of the chief pollinator, honey bee. We obtained 6,645 putative novel lncRNAs from 3,897 gene loci in the *V. destructor*, including 2,066 sense lncRNAs, 2,772 lincRNAs, and 1,807 lncNATs. The characteristics of the lncRNAs and the comparison with mRNAs were also reported. To verify the reliability of the RNA-seq results and the predicted lncRNAs, 16 non-coding transcripts were randomly selected to do RT-PCR validation, and 93.8% lncRNAs amplified signal bands denoting a reliable output of lncRNAs identified in this study. To the best of our knowledge, lncRNA studies have not performed in the parasitiformes species before.

*V. destructor* reproduction is limited to a short window when the immature honey bee host is concealed in wax cells. Mated female mites target to meal on the larva host 5 h after the invaded cell was capped (Ifantidis, 1988; Donzé and Guerin, 1994) and initiates oogenesis about another hour later (Garrido et al., 2000). The mite then lays the first male egg approximately 70 h after the cell capping (Donzé and Guerin, 1994; Martin, 1994). Considering these timing, we collected the mites 48 h later after the mite was introduced into the freshly capped cells. The artificial infestation has been established to be a suitable method for *V. destructor* research (Dietemann et al., 2013; Lin et al., 2018; Häußermann et al., 2019), and mites parasitized in the capped brood cells are less variable in physiology and fitness than on the adult bee bodies (Milani, 1995). The mites collected were further confirmed gravid with dilated post-abdomen, from which we can even see the eggs inside under microscope (Supplementary Figure 1B).

Just as lncRNA screening in other species, lincRNAs, the most extensively studied category of lncRNAs, usually account for the largest proportion (Sun et al., 2013; Wu et al., 2016; Zhan et al., 2016; Guo et al., 2018a), although some studies show otherwise (Guo et al., 2018b). We did not detect any ilncRNAs, which were regarded as the lowest conservative class of lncRNAs (Wang et al., 2016), in *V. destructor*. Intriguingly, this is also the case of *N. ceranae*, another pathogenic agent of honey bees (Guo et al., 2018b). Most of the *V. destructor* lncRNAs contained two exons with the average of 3.0 exons, significantly less than mRNAs (Figure 2 and Supplementary Table 2), which was also in line with the related studies of lncRNAs on other invertebrate counterparts, even mammalian and plant species (Trapnell et al., 2010; Zhang et al., 2014; Wu et al., 2016; Zhan et al., 2016; Guo et al., 2018a,b; Liu et al., 2019). Besides, the shorter length of the putative lncRNAs and the ORFs compared to mRNA also shared similar features with other well-studied species (Trapnell et al., 2010; Zhang et al., 2014; Wang et al., 2016; Wu et al., 2016; Zhan et al., 2016; Guo et al., 2018a,b; Liu et al., 2019). Although lncRNAs always performed lower expression level than the protein-coding genes (this study; Guo et al., 2018a,b; Chen et al., 2019; Liu et al., 2019), their role in the functional activities of organisms has been widely proved to be of significance (Furuno et al., 2006; Mercer et al., 2009; Zhang et al., 2014; Wu et al., 2016; Chen et al., 2019; Liu et al., 2019).

LncRNAs can operate in *cis* to regulate the transcriptional expression of neighboring genes on the same allele (Robinson et al., 2020). The upstream lncRNAs with intersection of promoter or other *cis* elements may regulate gene expression at the level of transcription or post-transcription, and lncRNAs in the downstream or 3’UTR region may have other regulatory functions. LncRNAs in less than 100 kb up/down stream of a gene may serve as *cis* regulatory factors (Guil and Esteller, 2012; Feng et al., 2019). The *cis* target genes were engaged in various molecular functions and biological processes. GO and KEGG analyses revealed the target genes were mainly included in protein binding, enzyme activities, metabolism, signaling molecules and interaction, and so forth (Figure 4 and Supplementary Tables 3, 4). Intriguingly, 25 genes were enriched in a pathway of dorso-ventral axis formation (Figure 4B and Supplementary Table 4), suggesting that they may play a crucial role in the development and regeneration during the process of oogenesis of the mite.

Similar to proteins, lncRNAs are of importance to be localized in specific cellular compartments, which provides insights for understanding their complex biological functions (Chen, 2016). We predicted the subcellular localizations of the identified sense lncRNAs, lincRNAs, and lncNATs, of which 95.6, 91.7, and 92.4% were respectively accumulated in nucleus (Figure 3). These lncRNAs have been proposed to play strong roles in nuclear architecture and gene expression regulation. They are associated with chromatin-modifying complexes, directly influence transcription, act as precursors for small RNAs, participate in stem cell pluripotency and differentiation, and so forth (Chen and Carmichael, 2010). Cytoplasmic lncRNAs, the second most popularly located lncRNAs, have been evidenced to impact gene expression in a variety of ways, such as interfering with protein post-translational modifications with a result of aberrant signal transduction (Lin et al., 2016), acting as decoys for miRNAs and proteins (Cesana et al., 2011; Lee et al., 2016) and affecting mRNA translation in the cytoplasm (Gong and Maquat, 2011). Hence, the lncRNAs in distinct subcellular compartments are of great interest to decipher their diverse functional significance.

## Conclusion

*V. destructor* presents tremendous threat to apiculture worldwide, and the in-depth molecular studies on the parasite will facilitate to control this unpopular pest. We reported the lncRNA profile of *V. destructor* by genome-wide RNA-seq in this study, and the genomic and structural features of the lncRNAs showed consistent with their counterparts in other species. Evidence is becoming increasingly clear that the function of lncRNAs is associated with their unique subcellular localization (Chen, 2016), and most of the lncRNAs detected in *V. destructor* were accumulated in nucleus. The target genes of lncRNAs were inferred to participate in diverse regulatory functions via *cis* regulation by GO term and KEGG pathway enrichment analyses. Our data provide genetic resources for exploration of the functional roles of lncRNAs involved in the ectoparasite *V. destructor*. Further studies would be of interest and value to characterize the expression profile of lncRNAs in the different life stages of the ubiquitous mite.

## Data Availability Statement

The datasets generated for this study can be found in the online repositories. The names of the repository/repositories and accession number(s) can be found below: https://www.ncbi.nlm.nih.gov/genbank/, SRA accession: SRP258850.

## Author Contributions

TJ, GC, and ZL conceived the ideas and designed the study. YBL, XC, and CH carried out the experimental infestations. ZL, WW, YK, XS, and YJL performed the data analyses. YBL, HC, and HX performed the validation experiment. ZL led the writing of the manuscript. All authors contributed critically to the drafts and gave final approval for publication.

## Conflict of Interest

The authors declare that the research was conducted in the absence of any commercial or financial relationships that could be construed as a potential conflict of interest.

## Footnotes

1 https://www.ncbi.nlm.nih.gov/assembly/GCF_002443255.1

## Supplementary Material

Supplementary Figure 1 The dorsal **(A)** and post-abdomen **(B)** appearances of the gravid adult female *Varroa destructor* we collected.

Supplementary Figure 2 Distribution percentage of the clean reads from Vd-1 **(A)**, Vd-2 **(B)**, and Vd-3 **(C)** mapped to the genome regions of *Varroa destructor*.

Supplementary Figure 3 RT-PCR validation of the randomly selected 16 lncRNAs. DNA marker (M) were used to indicate the product size in the left lane. Lane 1 to lane 16 were 16 lncRNAs orderly listed in the Supplementary Table S1.

Supplementary Table 1 Primer sets used for RT-PCR validation of the identified lncRNAs.

Supplementary Table 2 Information of all the transcripts.

Supplementary Table 3 Gene ontology (GO) output for the *cis* target genes of the identified lncRNAs.

Supplementary Table 4 Kyoto encyclopedia of genes and genomes (KEGG) pathway output for the *cis* target genes of the identified lncRNAs.

Supplementary Table 5 Sanger sequencing results of the 13 lncRNAs.
