# Supplementary material for: Genome-Wide Identification of Long Non-coding RNAs in the Gravid Ectoparasite Varroa destructor
Source: Front Genet. 2020 Oct 16;11:575680. doi: 10.3389/fgene.2020.575680 (PMC7596327; doi:10.3389/fgene.2020.575680)
Supplement: Supplementary Figure 1 — The dorsal (A) and post-abdomen (B) appearances of the gravid adult female Varroa destructor we collected. [file Data_Sheet_1.zip › Supplementary Tables & Figures.PDF]

Supplementary Material

Genome-wide identification of long non-coding RNAs in the gravid ectoparasite *Varroa destructor*

Zheguang Lin[1], Yibing Liu[1], Xiaomei Chen[1], Cong Han[1], Wei Wang[2], Yalu Ke[2], Xiaoling Su[3], Yujiao Li[4], Heng Chen[1], Hao Xu[1], Guohong Chen[1], Ting Ji[1,*]

[1] Apicultural Research Institute, College of Animal Science and Technology, Yangzhou University, Yangzhou 225009, P.R. China

[2] Wuzhong Animal Health Supervision Institute, Suzhou 215100, P.R. China

[3] Jinhua Academy of Agricultural Sciences, Jinhua 321017, P.R. China

[4] Shandong Apiculture Breeding of Improved Varieties and Extension Center, Taian 271000, P.R. China

*Correspondence:

tji@yzu.edu.cn

Supplementary information includes:

Table S1

Table S2 – S5 (in a separate Excel)

Figure S1 – S3

Table S1. Primer sets used for RT-PCR validation of the identified lncRNAs.

Novel lncRNA ID	Primer type	Sequence	PCR product size (bp)
TCONS_00047157	Forward	AGTGAATGCGGACGAAGATGAGTG	305
	Reverse	GATAACACTGGCTGGCGGTCATAC	
TCONS_00053275	Forward	CGCCACCGAAGGTTGCTGATAG	317
	Reverse	GCGTCTAACAGCGAGTCACCTATC	
TCONS_00030248	Forward	CGCCAAATCCTTGAAGTTCAGCAG	229
	Reverse	GCCACACTCGGCACGGTATTG	
TCONS_00117995	Forward	CTGAGGGAAACTTCGGAAGGAACC	361
	Reverse	CTGCGAGTGAACTGGAACTAAGCC	
TCONS_00096863	Forward	TCTCTGTCGTCGTCAGGTGTCC	381
	Reverse	GGCTGTCCTTTCCGCTAGTGTTTC	
TCONS_00116783	Forward	CGGACTGCCACCAAGATAATCACC	233
	Reverse	TGCACACTGGGAGGCTACTTAGG	
TCONS_00116781	Forward	TCGGGTCTCAAACATCACGCTTG	223
	Reverse	ACTCTAGGTAAGCAGTCCGCCATC	

TCONS_00085392	Forward	GAGCGGAATGAATTTGGCAGAAGC	359
	Reverse	GGCTATGCGTGGTTCTCCTTTGG	
TCONS_00079678	Forward	CGTCGATTACTGTGAGGCCAAGC	320
	Reverse	TGCGGTAAACTCGTCAATGGTGTC	
TCONS_00116724	Forward	GTGCTGTAGAGTGGTGTGCCTATC	203
	Reverse	TGACGCCCAACACAGAACATCG	
TCONS_00116766	Forward	TTAGGACCGACTGACCCACGATC	268
	Reverse	ACTCACCTGCCAAAGCAACTAGC	
TCONS_00116746	Forward	TGATGCCTTGAGCGTAAGTTGTCC	385
	Reverse	TTCACTTCGCCTTCGGGTGTTAAG	
TCONS_00116726	Forward	ATCGTGGGTCAGTCGGTCCTAAG	364
	Reverse	ACCTTCAGAGCCATCCCTTCTCC	
TCONS_00116735	Forward	TGCACACTGGGAGGCTACTTAGG	233
	Reverse	CGGACTGCCACCAAGATAATCACC	
TCONS_00116712	Forward	TGCTGTAAAGTGGTGTGCTAGTGC	236
	Reverse	TGATGGCGGACTGCTTACCTAGAG	
TCONS_00117418	Forward	TGGTGCCTTGAGCGTAAGTTGTC	288
	Reverse	TGATGGCGGACTGCTTACCTAGAG	

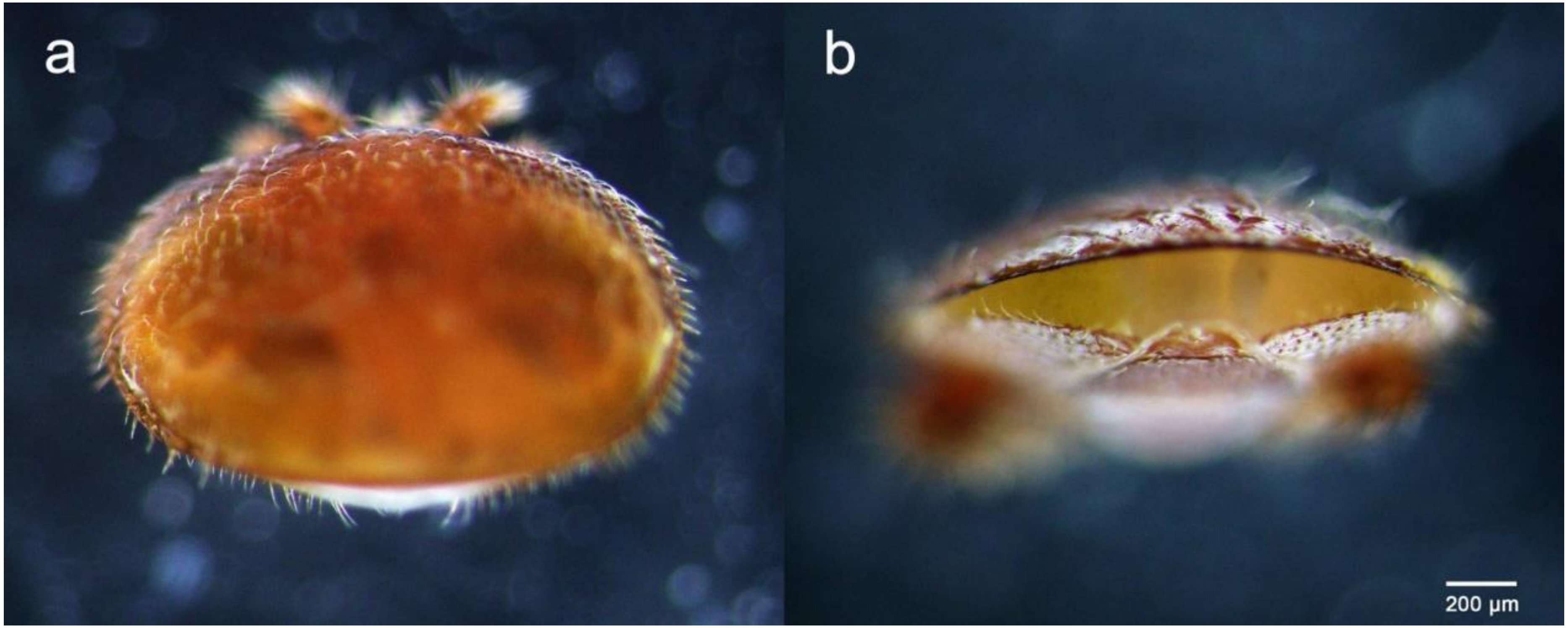

Figure S1. The dorsal (**a**) and post-abdomen (**b**) appearances of the gravid adult female *Varroa destructor* we collected.

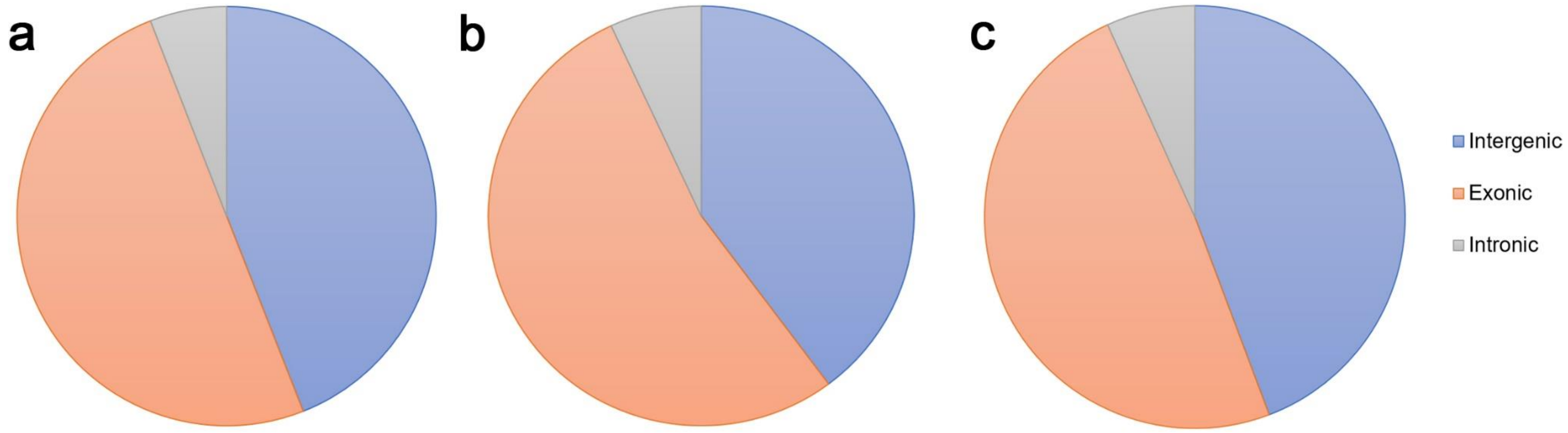

Figure S2. Distribution percentage of the clean reads from Vd-1(**a**), Vd-2(**b**) and Vd-3(**c**) mapped to the genome regions of *Varroa destructor*.

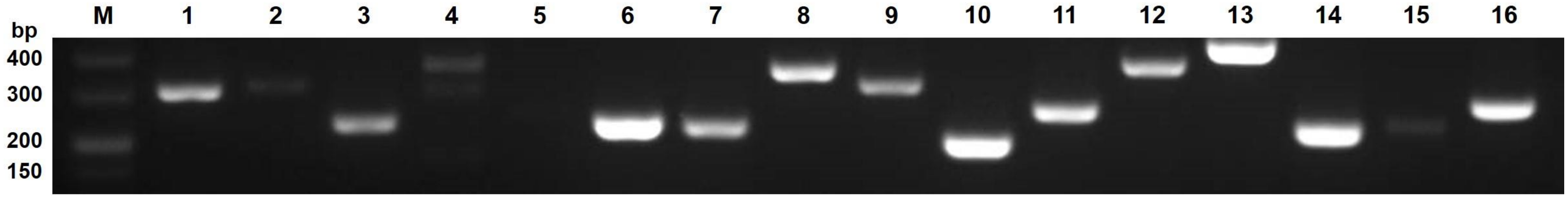

Figure S3. RT-PCR validation of the randomly selected 16 lncRNAs. DNA marker (M) were used to indicate the product size in the left lane. Lane 1 to lane 16 were 16 lncRNAs orderly listed in the Table S1.
